# Size-Dependent Mechanical Properties of Additively Manufactured Ti-6Al-4V Thin Walls

**DOI:** 10.3390/ma19101945

**Published:** 2026-05-09

**Authors:** Tenglong Xie, Chao Ding, Peng Wang, Minghao Huang, Shenghang Xu, Zhen Wang, Huiping Tang

**Affiliations:** 1 Zhejiang Key Laboratory of Aerospace Metallic Materials, Hangzhou City University, Hangzhou 310015, China; tenglongxie2023@163.com (T.X.);; 2 School of Civil Engineering, Shaoxing University, Shaoxing 312000, China

**Keywords:** laser powder bed fusion, Ti-6Al-4V thin walls, size effect, mechanical properties, printing defects

## Abstract

Additively manufactured lightweight lattice structures typically consist of thin walls with thicknesses ranging from hundreds of micrometers to millimeters. Within this range, such thin walls exhibit a pronounced size effect. Despite extensive research on the topic, a clear mapping between key influencing factors and mechanical properties remains lacking. This gap makes it challenging to accurately predict mechanical performance across different wall thicknesses, especially for those below 500 μm. In this work, Ti-6Al-4V thin-walled tensile specimens with thicknesses ranging from 0.2 mm to 1.0 mm were fabricated via laser powder bed fusion (LPBF). The variations in mechanical properties, microstructure, surface defects, and internal defects were investigated. The results indicate that yield strength (YS) and ultimate tensile strength (UTS) decreased significantly as thickness decreased, dropping from 794.1 MPa to 471.7 MPa and from 910.7 MPa to 485.2 MPa, respectively. Printing defects were identified as the dominant factors governing the size effect: strength was jointly affected by surface and internal defects, whereas failure mode and ductility were primarily governed by internal defects. By introducing an effective thickness ratio parameter, a semi-empirical predictive model was developed to characterize the strength-thickness relationship and to quantify the individual contributions of surface defects and other coupled interior factors. Subsequently, ultra-thin specimens were subjected to surface grinding and polishing to alleviate surface defects, leading to improvements in YS and UTS of approximately 27–39% and 22–45%, respectively. The model-predicted strengths of the surface-treated specimens were in good agreement with the measured values, further validating the effectiveness of the proposed model.

## 1. Introduction

Metallic lattice structures exhibit high specific strength, superior damping, excellent energy absorption, and efficient heat transfer, making them widely used in aerospace, automotive engineering, biomedicine, and beyond [1,2,3,4]. Among the materials for such structures, Ti-6Al-4V alloy has become one of the most widely used metallic matrices, owing to its high specific strength, corrosion resistance, and favorable biocompatibility [5,6]. Recently, advances in additive manufacturing, particularly LPBF [7,8,9], have enabled more precise design and tailoring of Ti-6Al-4V lattices, thus enhancing their comprehensive performance [10,11,12].

As lattice structures evolve toward lighter weight and finer features, they increasingly incorporate thin-walled elements with thicknesses ranging from hundreds of micrometers to millimeters [13,14,15,16]. Such thin-walled structures achieve high specific strength by converting external loads into membrane stresses, thereby improving load-bearing efficiency. However, substantial size effects within this thickness range can significantly influence their mechanical performance, manifesting as a competition between two counteracting effects: strengthening [17] and weakening [18]. The strengthening effect originates from microstructural refinement and chemical changes. As thickness decreases, the microstructure of thin walls gradually refines, leading to material strength enhancement [17,19,20]. Simultaneously, thinner specimens exhibit higher surface-to-volume ratios, promoting oxygen diffusion into the material during printing and heat treatment [17]. Oxygen can effectively improve the strength of titanium alloys, though often at the expense of reduced ductility [21,22]. Conversely, the weakening effect is governed by surface quality and internal defects [18,23,24]. LPBF-fabricated components typically exhibit rough surfaces and internal porosity resulting from printing-induced issues such as incomplete melting, balling effects, and powder spattering [25,26,27]. As wall thickness decreases, surface roughness and internal porosity tend to intensify, thereby amplifying their detrimental impact on mechanical properties [28]. Specifically, incompletely melted powder particles adhering to the surface lack load-bearing capacity, which reduces the effective load-bearing cross-section [29]. In addition, both surface defects and internal pores promote premature structural failure by inducing stress concentrations [28,30,31]. These combined factors result in a pronounced reduction in strength and ductility with decreasing thickness. When the thickness falls below 1 mm, printing defects, notably surface roughness and internal porosity, become increasingly dominant. Although microstructural refinement still provides a certain strengthening effect, its beneficial contribution is outweighed by defect-induced mechanical degradation, resulting in an overall decline in performance. Zhang et al. [18], for instance, reported a nearly 20% reduction in strength for LPBF-fabricated Ti-6Al-4V thin walls as the thickness decreased from 2.0 mm to 0.5 mm. This tendency was even more pronounced in components produced by electron beam powder bed fusion (EBPBF), where the tensile strength of 0.5 mm thick specimens retained only approximately 50% that of 4.0 mm thick ones, due to more severe printing defects in the EBPBF process [32]. While post-processing techniques such as grinding and polishing can help mitigate such degradation [23,33,34,35], they are often impractical for intricate thin-walled lattice structures. Therefore, it remains crucial to clarify the correlation between thickness and the mechanical properties of as-printed thin walls for reliable and accurate structural design.

Due to the complexity of multi-factor coupling effects, current research remains largely focused on identifying various influencing factors and characterizing their positive or negative impacts on the mechanical properties of thin walls. Quantitative assessment of the relative contribution of each factor, however, is still limited. Consequently, in practical applications, accurate prediction of mechanical performance with thickness remains challenging [36]. To address this, some studies employed 3D scanning techniques to reconstruct digital models of as-printed thin-walled structures. These models accurately captured the detailed features of surface and internal defects. Based on these models, finite element simulations were then performed to evaluate the mechanical response under the influence of printing defects [37,38,39]. However, the geometric complexity involved often makes these simulations computationally expensive, particularly when applied to structures composed of numerous thin-walled elements, such as lattice structures. An alternative method is to establish empirical fitting equations relating thickness to mechanical properties based on experimental data [36]. This approach allows for rapid estimation of mechanical properties across various thicknesses with minimal computational cost. Nevertheless, its accuracy depends on the selection of fitting functions, and it lacks a clear physical basis, making it difficult to further quantify the individual contributions of different factors. More importantly, most studies on the size effect in Ti-6Al-4V thin walls focused on thicknesses above 0.5 mm. The mechanical behavior of ultra-thin walls in the range below 0.5 mm, which approaches the manufacturing limit, remains unclear [19]. It is uncertain whether empirical models derived from thicker walls can be extended to this ultra-thin regime, which limits their guidance for the design of advanced lightweight thin-walled structures.

**Figure 1:**
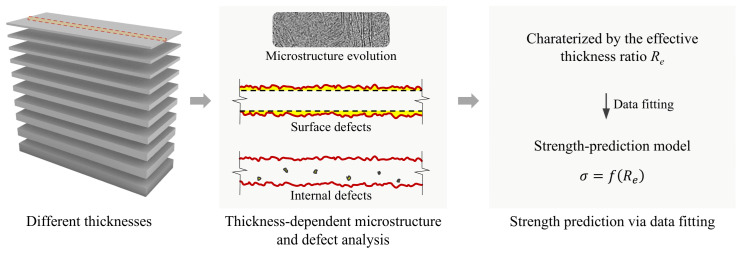
Schematic workflow of this work.

To address these challenges, this work systematically investigated the size-dependent mechanical properties of LPBF-fabricated Ti-6Al-4V thin walls with thicknesses ranging from 0.2 mm to 1.0 mm. Tensile tests were conducted to examine the variation in strength, elongation, and failure modes with respect to wall thickness. As illustrated in the schematic workflow (Figure 1), the microstructure, surface morphology, and internal defects of thin-walled specimens across different thicknesses were characterized to reveal their evolution and influence on mechanical behavior. Building on these observations, the effective thickness ratio was introduced to quantitatively evaluate the effects of these factors. Combined with experimental data fitting, a semi-empirical strength-prediction model was established for the thin walls with different thicknesses. This model enables quantification of the individual contributions of surface defects and other coupled interior factors. Finally, by comparing the tensile properties of specimens before and after surface grinding, the proposed predictive model was validated by isolating the specific effect of surface defects.

## 2. Materials and Methods

### 2.1. Specimen Fabrication

This work aims to investigate the size effect over a typical thickness range from 0.2 mm to 1.0 mm, which is widely adopted in lightweight thin-walled lattice structures. Accordingly, tensile specimens were fabricated at 0.1 mm increments across this range, resulting in nine discrete thickness levels. To better capture the pronounced size effect expected in the ultra-thin regime of 0.2–0.3 mm, an additional specimen with a thickness of 0.25 mm was included. In total, specimens of ten different thicknesses were fabricated via LPBF. For each thickness, at least five replicas were produced, of which three were subjected to tensile testing, and the remaining two were reserved for defect analysis and microstructural characterization. The limited sample size (*n* = 3) for tensile testing was chosen on an exploratory basis. Therefore, the tensile results should be interpreted as preliminary observations rather than definitive population estimates.

**Figure 2:**
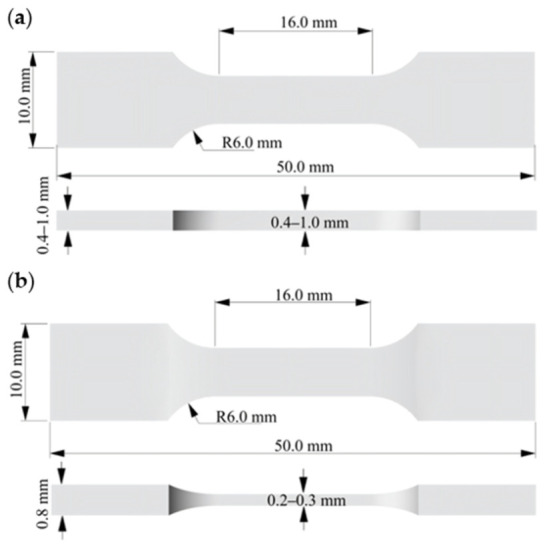
Shape and dimensions of the tensile specimens: (**a**) thickness of 0.4–1.0 mm; (**b**) thickness of 0.2–0.3 mm.

The specimen geometry is detailed in Figure 2, featuring an overall length of 50 mm, a parallel segment length of 16 mm, and widths of 6 mm in the gauge section and 10 mm at the gripping ends. Most dimensions were scaled from those specified in the ASTM E8/E8M-13a standard [40]. For the ultra-thin specimens (0.2–0.3 mm), the gripping ends were locally thickened to 0.8 mm with a filet to ensure a smooth thickness transition (Figure 2a), primarily to prevent slippage or damage during testing. This geometric modification was considered to exert a negligible influence on the tensile results [36]. Experimental observations also confirmed that failure of the ultra-thin specimens occurred predominantly within the gauge section, away from the gripping ends, which suggested that the influence of the local geometric modification was likely limited. In contrast, for specimens with thicknesses of 0.4 mm and above, a uniform-thickness design proved sufficient for stable gripping and was therefore retained (Figure 2b).

The tensile specimens were fabricated using LPBF. Argon-gas-atomized Ti-6Al-4V alloy powder, supplied by Bright Laser Technologies (Xi’an, China), was employed. The particle size distribution was characterized using a laser diffraction particle size analyzer (Malvern Mastersizer 3000, Malvern Panalytical Ltd., Malvern, UK). The results show that *D*_10_ = 27.0 μm, *D*_50_ = 42.3 μm, and *D*_90_ = 56.5 μm. The chemical composition of the powder was measured via inductively coupled plasma (ICP) analysis, with detailed results summarized in Table 1.

Specimen printing was performed using a BLT-A300 machine (Bright Laser Technologies, Xi’an, China). The process parameters were set as follows: a laser power of 190 W, a layer thickness of 30 μm, a scan speed of 90 mm/s, and a hatch spacing of 140 μm. Upon completion, the samples were naturally cooled to room temperature in the argon-filled build chamber before removal. They were then detached from the substrate via wire electrical discharge machining and ultrasonically cleaned in ethanol for 15 min. As this study specifically investigates the size effect arising from the thickness variation, all specimens were printed in the identical orientation to avoid interference from the build direction.

### 2.2. Tensile Experiment

**Figure 3:**
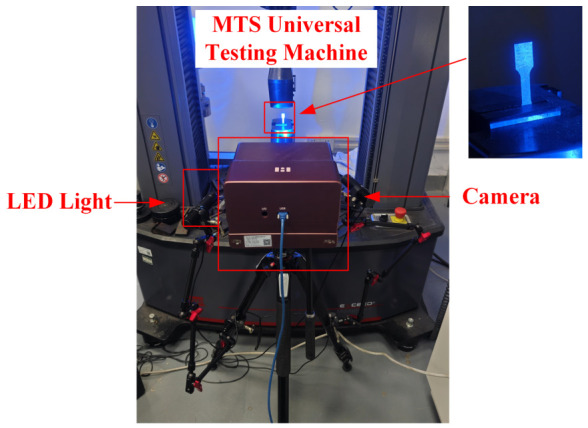
Tensile testing setup.

As shown in Figure 3, tensile tests were performed using an MTS universal testing machine (MTS Systems Corporation, Eden Prairie, MN, USA) at a loading rate of 0.7 mm/min. To accurately capture deformation during loading, a Digital Image Correlation (DIC) system was employed for displacement field measurement. Prior to testing, the gauge section of each specimen was uniformly coated with a matte white primer, after which black speckles with diameters ranging from 0.2 to 0.4 mm were randomly applied. This procedure produced a high-contrast and randomly distributed speckle pattern suitable for DIC analysis. Throughout the loading process, speckle images of the specimen surface were recorded synchronously by a camera at a sampling rate of 4 frames per second (fps). Following the tests, the acquired image sequences were processed using VIC-3D software (Version 11.2, Correlated Solutions, Inc., Irmo, SC, USA) to obtain full-field displacement data and strain distributions within the gauge section.

### 2.3. Microstructure Characterization

To investigate the microstructural features across varying thicknesses, metallographic samples were prepared from the gauge sections. After standard grinding and polishing, the samples were lightly etched with Kroll’s reagent (2 mL of HF, 4 mL of HNO_3_, and 94 mL of H_2_O) to reveal the microstructure. Backscattered electron (BSE) imaging was subsequently performed using a scanning electron microscope (Thermo Scientific Scios 2, Thermo Fisher Scientific Inc., Waltham, MA, USA).

### 2.4. Defect Characterization

To characterize the surface condition, each tensile specimen was examined using a digital microscope (Leica DVM6 M, Leica Microsystems GmbH, Wetzlar, Germany) prior to testing. The specimens were oriented with their thickness directions facing the lens. The gauge section was imaged and measured at discrete intervals, and the acquired images were then stitched to reconstruct its complete surface topography.

Additionally, to analyze internal defects, metallographic samples were extracted from the gauge sections of specimens with different thicknesses. After standard grinding and polishing, these samples were imaged using the same digital microscope to characterize the distribution of internal pores.

For both surface and internal defect observations, the digital microscope was set to a resolution of 1073 lp/mm and a magnification ranging from 150 to 400, which enabled clear identification of micron-scale powder particles adhering to the specimen surface and of internal pores in the metallographic samples.

## 3. Results and Discussion

### 3.1. Mechanical Properties

**Figure 4:**
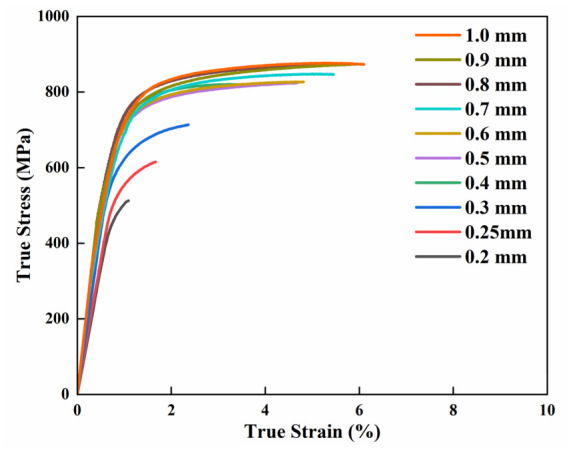
Stress–strain curves of specimens with various thicknesses.

The tensile stress–strain curves for the specimens of different thicknesses are presented in Figure 4. True stress (*σ_true_*) and strain (*ε_true_*) were converted from engineering stress (*σ_eng_*) and strain (*ε_eng_*) using the following relations:

(1) $$\sigma_{true}=\sigma_{eng}\left(1+\varepsilon_{eng}\right),\;\varepsilon_{eng}=ln\left(1+\varepsilon_{eng}\right)$$

Engineering strain was derived from the DIC measurement system, while engineering stress was calculated from the measured load as

(2) $$\sigma_{eng}=\frac{F_{tensile}}{A_{a}}$$

where *F_tensile_* is the tensile force, and *A_a_* denotes the apparent cross-sectional area within the gauge region, which is determined as the product of the apparent thickness and the apparent width.

**Figure 5:**
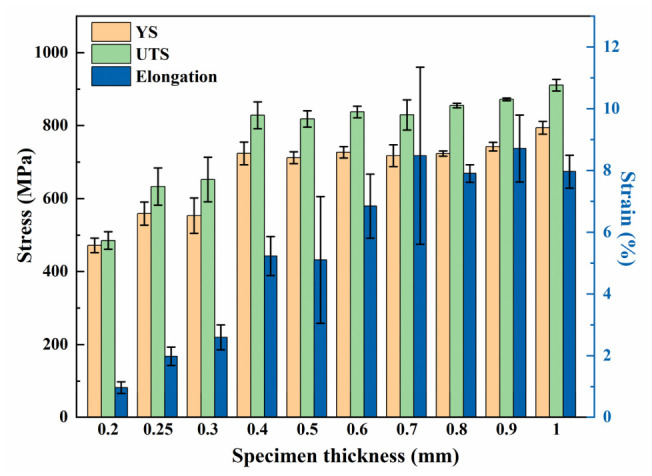
Mechanical properties of specimens with various thicknesses.

The corresponding mechanical properties, i.e., YS, UTS, and elongation, are exhibited in Figure 5, where the YS values were determined using the 0.2% offset method. To ensure reproducibility, three replicate specimens were tested for each thickness condition. The small standard deviations, shown as error bars in Figure 5, indicate good consistency among the replicates. This supports the statistical reliability of this exploratory study despite the limited sample size.

The experimental results demonstrated that increasing the specimen thickness from 0.2 mm to 1.0 mm led to a significant enhancement in mechanical properties. YS increased markedly from 471.7 ± 19.6 MPa to 794.1 ± 17.5 MPa, while UTS rose from 485.2 ± 24.1 MPa to 910.7 ± 15.6 MPa. Meanwhile, the elongation improved from 1.2 ± 0.3% to 8.5 ± 0.5%, collectively revealing a distinct size effect.

In the ultra-thin regime (0.2–0.3 mm), strength increases substantially with thickness. Despite a marginal improvement, the elongation remains consistently below 3%, suggesting predominantly brittle fracture behavior. In contrast, for thicknesses ranging from 0.4 mm to 1.0 mm, the strength plateaus with only a marginal further increase. Within this range, ductility is markedly enhanced compared to the ultra-thin specimens, reaching approximately 5% at 0.4–0.5 mm and exceeding 6% at 0.6 mm and above.

**Figure 6:**
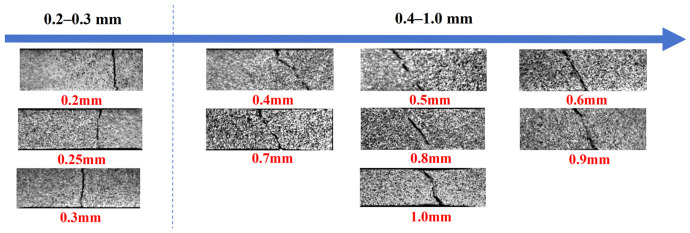
Fracture morphology of specimens with various thicknesses.

Figure 6 presents the fracture morphology across different thicknesses. The ultra-thin specimens (0.2–0.3 mm) exhibit transverse fractures perpendicular to the tensile direction, whereas those with thicknesses of 0.4 mm and above fail by shear fractures along the direction of maximum shear stress. This transition in fracture mode aligns with the distinct contrast in ductility between the two thickness regimes.

**Figure 7:**
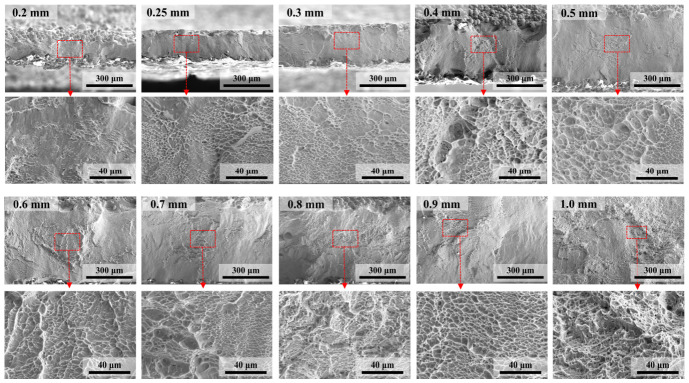
Cross-sectional fracture morphologies of specimens with various thicknesses after tensile testing. For each thickness, the lower micrograph shows a high-magnification view of the red-boxed region in the corresponding upper micrograph.

To further investigate the failure modes across varying thicknesses, the cross-sectional fracture morphologies after tensile testing are presented in Figure 7. For the 0.2 mm specimen, the fracture surface is characterized by extensive cleavage-like facets, signifying a typical brittle fracture mode. In contrast, the 0.25 mm and 0.3 mm specimens predominantly exhibit dimple features. However, these dimples are relatively shallow and localized, which is consistent with their limited macroscopic plasticity. As the thickness increases to 0.4 mm and beyond, the dimples become markedly deeper, indicating a transition toward a more ductile fracture mechanism and a substantial improvement in elongation.

The experimental results reveal a pronounced size effect on strength, ductility, and fracture behavior. The underlying mechanisms of this effect are discussed below in terms of three key factors: microstructure, surface defects, and internal defects.

### 3.2. Microstructure

**Figure 8:**
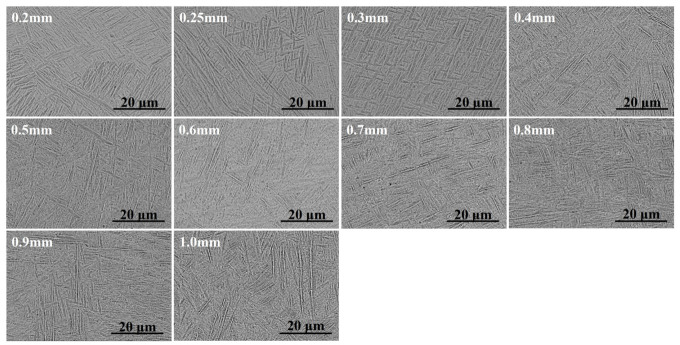
BSE images of the specimen surfaces across various thicknesses.

Figure 8 shows the BSE images of specimen surfaces with various thicknesses, revealing typical thin α′ martensitic laths across all specimens. To quantitatively assess microstructural variations, fifty α′ laths were randomly selected and measured for each specimen. The average lath width and length are summarized in Table 2, and the detailed information on the measurement regions and raw data are provided in Figure S1 and Table S1. The α′ laths exhibit slight refinement as specimen thickness decreases. This trend is consistent with previously reported findings [18,20], in which the microstructures, including both α′ laths and prior β grains, refined progressively with decreasing thickness, yet the extent of such microstructural changes was limited.

In principle, finer α′ laths can hinder dislocation motion and thus produce a strengthening effect. Nevertheless, this strengthening contribution is expected to be relatively small due to the limited degree of microstructural refinement. On this basis, thinner specimens with slightly refined α’ laths are expected to exhibit slightly higher strength. This theoretical expectation, however, is inconsistent with the experimental observations, in which both YS and UTS decreased markedly with decreasing thickness (with a maximum decrease of over 400 MPa). This indicates that the beneficial effect of microstructural refinement is completely outweighed by the much more pronounced weakening effect induced by surface and internal defects in thinner specimens.

### 3.3. Surface Defects

**Figure 9:**
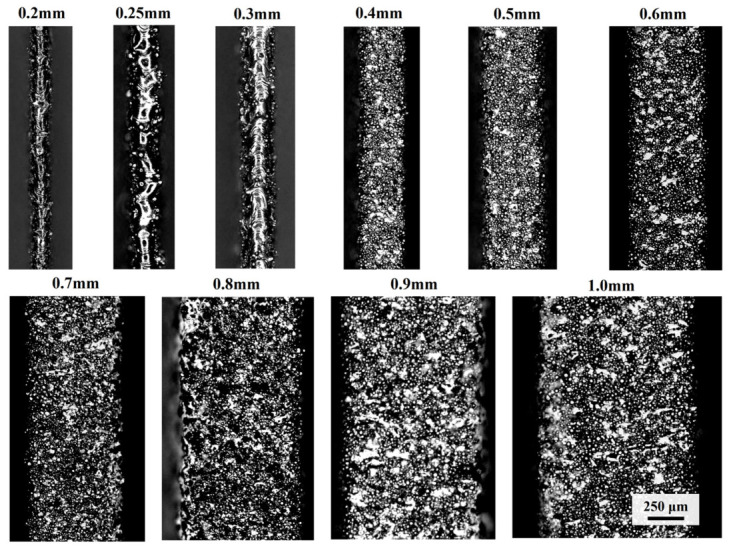
Surface topography images of as-printed thin-walled specimens.

Surface topography images of as-printed thin walls at various thicknesses are presented in Figure 9. All specimens show high surface roughness with adherent unmelted particles. The reduction in thickness leads to a higher cooling rate, which may inhibit adequate stabilization and spreading of the melt pool. This, in turn, induces a pronounced balling effect in the ultra-thin specimens with thicknesses of 0.2–0.3 mm.

**Figure 10:**
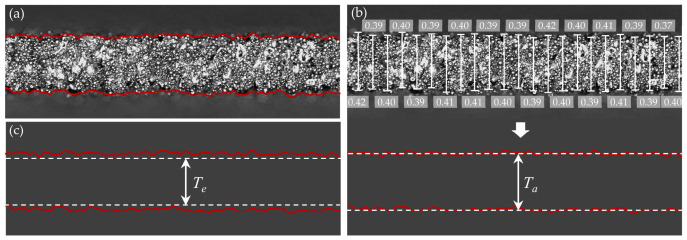
Schematic diagrams of specimen thickness characterization: (**a**) outer contour extraction; (**b**) apparent thickness and its measurement method; (**c**) effective thickness.

Thickness profiles were generated by stitching the acquired images and extracting the external contours, as shown in Figure 10a (only a short segment is displayed for clarity). The thickness along the gauge section was then measured from these profiles. The apparent thickness (*T_a_*), used to calculate the apparent stress, is defined as the average distance between the outer contours, as illustrated in Figure 10b. It was determined by measuring the thickness at intervals of approximately 150 μm along the gauge length using a digital microscope, and then averaging the measured values. The effective thickness (*T_e_*), shown in Figure 10c, represents the thickness of the continuous load-bearing region within the outer contours. It corresponds to the maximum distance between two parallel lines that can be drawn within the contour without intersecting the outer boundary, thereby excluding the rough surface layer that lacks load-bearing capacity. The effective thickness ratio is then given by *R_e_* = *T_e_*/*T_a_*. All thickness data obtained from this image analysis are listed in Table 3.

As shown in the table, the apparent thickness of each specimen closely matches its design value, with a maximum deviation of approximately 50 μm, demonstrating high dimensional precision of the printing process. Notably, the effective thickness ratio exhibits a pronounced increase within the 0.2–0.4 mm thickness range, then gradually plateaus beyond 0.4 mm. This trend is consistent with variation in specimen strength, revealing a strong positive correlation between these parameters. As thickness increases, the relative proportion of the defective surface layer decreases, thereby progressively reducing its detrimental effect on mechanical properties.

Denoting the thickness of the defective surface layer on one side as *T_s_*, and accounting for the presence of such layers on both sides of the specimen, the effective thickness can be approximated as

(3) $$T_{e}=T_{a}-{2T}_{s}$$

Accordingly, the effective thickness ratio can be calculated as

(4) $$R_{e}=\frac{T_{e}}{T_{a}}=1-\frac{{2T}_{s}}{T_{a}}$$

**Figure 11:**
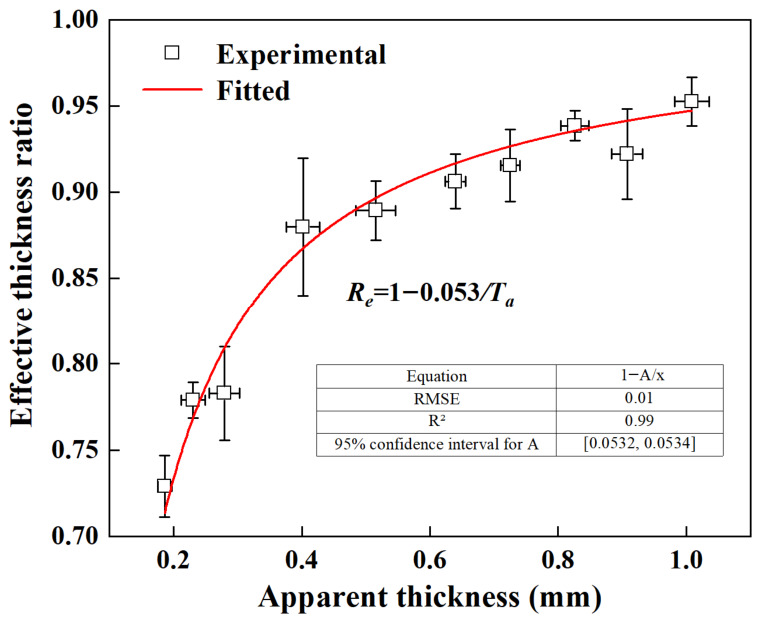
Correlation between effective thickness ratio and apparent thickness.

Figure 11 displays the correlation between the effective thickness ratio and the apparent thickness of the specimens. Fitting the data to Equation (4) gives a defective surface layer thickness (*T_s_*) of approximately 26.5 μm across all specimens. This result indicates that *T_s_* is independent of the overall specimen thickness and remains largely constant under consistent processing conditions. Therefore, according to Equation (3), the effective thickness ratio is inversely proportional to the specimen thickness. As thickness increases, the ratio approaches unity, indicating that surface defects become negligible in thicker specimens. In contrast, for thinner specimens, particularly those below 0.4 mm, surface defects account for a substantial portion of the cross-section and thus significantly reduce the mechanical properties. The quantitative impact of this effect will be discussed in Section 3.5.

### 3.4. Internal Defects

**Figure 12:**
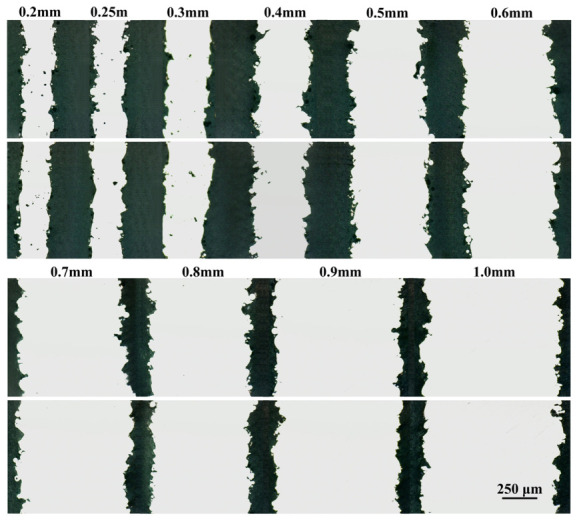
Micrographs of metallographic samples across various thicknesses.

Two metallographic samples were taken from the gauge section of each specimen for internal defect examination. Optical micrographs of these samples are presented in Figure 12. The size and area fractions of internal pores were quantified using ImageJ software (Version 1.54, National Institutes of Health, Bethesda, MD, USA), with the results summarized in Table 4. The micrographs clearly show incompletely melted powder particles and surface notches induced by the balling effect, in agreement with the observations in the previous section. In addition, significant internal porosity is observed in specimens with thicknesses between 0.2 and 0.3 mm. These pores are irregular in morphology and range from 2 to nearly 30 μm in size. Overall, porosity decreases markedly with increasing thickness, from 0.615% at 0.25 mm to only 0.018% at 1.0 mm. Notably, specimens of 0.4 mm and above show significantly lower porosity than those of 0.3 mm or below.

**Figure 13:**
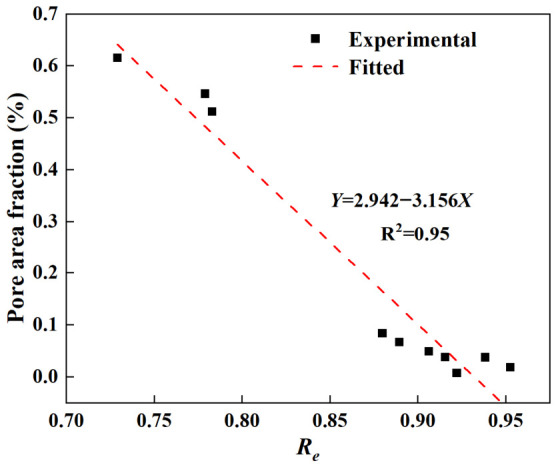
Relationship between pore area fraction and *R_e_*.

During the LPBF process, thinner walls exhibit higher cooling rates and steeper thermal gradients owing to limited heat-dissipation pathways. These conditions tend to destabilize the melt pool, promoting balling effects and surface irregularities [41,42]. As a result, ultra-thin specimens are more prone to defects involving both internal porosity and surface roughness. Since both types of defects originate from the same physical mechanism, they are highly correlated [43]. Figure 13 plots the relationship between the pore area fraction and the effective thickness ratio *R_e_*. An approximately linear trend is observed between these two parameters. Given that (1 − *R_e_*) represents the surface defect fraction, a strong negative correlation exists between pore area fraction and *R_e_*: thicker specimens correspond to larger *R_e_* values and lower porosity, whereas thinner specimens correspond to smaller *R_e_* values and higher porosity.

It should be noted that the two-dimensional measurements are limited to the inspected cross-sections and cannot fully represent the overall three-dimensional pore distributions. Each observed pore is only a planar slice of an irregular three-dimensional void, which may introduce stereological bias in area and size quantification. Consequently, the averaged pore fractions derived from these two-dimensional measurements should be regarded as approximate indicators, rather than precise three-dimensional volumetric values.

**Figure 14:**
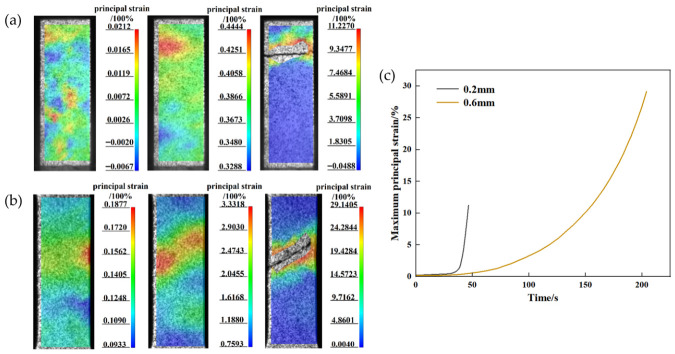
Strain distributions of the specimens: (**a**) 0.2 mm and (**b**) 0.6 mm; (**c**) maximum principal strain versus time for the two specimens.

The presence of internal porosity further reduces the effective load-bearing area and promotes stress concentrations, leading to degradation in mechanical performance. To further understand this impact, strain field evolution was analyzed in representative specimens with thicknesses of 0.2 mm and 0.6 mm. Their corresponding strain distributions during tensile loading were acquired via the DIC measurement system and are presented in Figure 14a,b. In the 0.2 mm specimen, strain concentrations appeared both internally and along the edges at the initial loading stage, due to the combined effects of surface defects and internal pores. As loading progressed, edge-localized strain propagated inward, coalescing with the internal strain concentration zone and ultimately forming a transverse fracture perpendicular to the loading direction. This interaction between surface and internal defects led to rapid failure with limited elongation in the ultra-thin specimens, corresponding to brittle fracture behavior. In contrast, the 0.6 mm thick specimen, which contained fewer internal defects, exhibited strain concentration primarily along the edges during early loading, while the internal strain distribution remained relatively uniform. With further loading, the strain concentration band extended along the direction of maximum shear stress, eventually resulting in a slant fracture. This failure mode is consistent with that of typical Ti-6Al-4V tensile specimens. Figure 14c shows the evolution of maximum principal strain over time for the two specimens. The 0.2 mm specimen exhibits a noticeably faster strain increase than the 0.6 mm specimen. Owing to the higher defect density, particularly internal porosity, the ultra-thin specimen exhibits much more severe early-stage strain localization. Consequently, its maximum strain increases steeply with applied load and reaches a high level early in the loading process. In contrast, the maximum strain in the thicker specimen increases gradually and steadily, indicating a more uniform internal strain distribution and weaker stress concentration. These contrasting strain evolution patterns indicate that internal defects are the primary factor governing the failure mode transition in thin-walled structures.

### 3.5. Size-Dependent Strength Prediction Model

Reducing specimen thickness refines the microstructure and induces a certain strengthening effect. Meanwhile, the increase in surface and internal defects produces a pronounced weakening effect. To evaluate the contribution of these factors, a semi-empirical model for size-dependent strength prediction is established in this section.

The influence of surface defects can be isolated and evaluated independently. However, strictly decoupling and quantifying the individual effects of internal defects, microstructural evolution, and oxygen content variation remains challenging. Accordingly, all influencing factors are divided into two groups: exterior factors corresponding to surface defects, and interior factors representing the coupled effects of internal defects, microstructural evolution, oxygen content variation, and other intrinsic contributions. These two categories of factors jointly govern the strength variation in thin-walled specimens. Their contributions are quantified by two coefficients, *η_s_* and *η_i_*, respectively. Assuming the theoretical strength of a defect-free Ti-6Al-4V material is *σ*_0_, the apparent strength in the presence of defects can be expressed as

(5) $$\sigma =\eta_{s}\eta_{i}\sigma_{0}$$

where *η_s_* and *η_i_* are the reduction factors for surface and coupled interior factors, respectively.

Given that the effective load-bearing thickness of the specimen is *T_e_*, the critical bearing capacity can be expressed in terms of the gauge width *W* as

(6) $$F=T_{e}W\eta_{i}\sigma_{0}$$

where *η_i_σ*_0_ represents the material strength when only interior factors are considered, with surface defects excluded.

Based on the critical bearing capacity, the apparent strength of the specimen is

(7) $$\sigma =\frac{F}{T_{a}W}=\frac{T_{e}}{T_{a}}\eta_{i}\sigma_{0}=R_{e}\eta_{i}\sigma_{0}$$

Comparing Equations (5) and (7) yields

(8) $$\eta_{s}=R_{e}$$

Equation (8) suggests that the strength reduction caused by surface defects directly equals the effective thickness ratio, *R_e_*.

Based on the above equation, *η_s_* decreases from 95.3% to 72.9% as the thickness is reduced from 1 mm to 0.2 mm. This corresponds to a reduction of approximately 23% in the strength induced by surface defects. Nevertheless, the measured strength exhibits a drop of more than 40%, indicating an additional weakening effect arising from the coupled interior factors. Since the interior factors include both detrimental internal defects and other beneficial intrinsic contributions, this net weakening effect confirms that the adverse impact of internal defects is dominant. Given the strong correlation between internal and surface defects as discussed earlier, their impacts on mechanical behavior are expected to follow similar trends. Accordingly, analogous to surface defects, the reduction coefficient for the coupled interior factors can also be expressed as a function of *R_e_* as an engineering simplification. Consequently, the apparent strength is simplified as a function of *R_e_*, which can be determined through data fitting.

**Figure 15:**
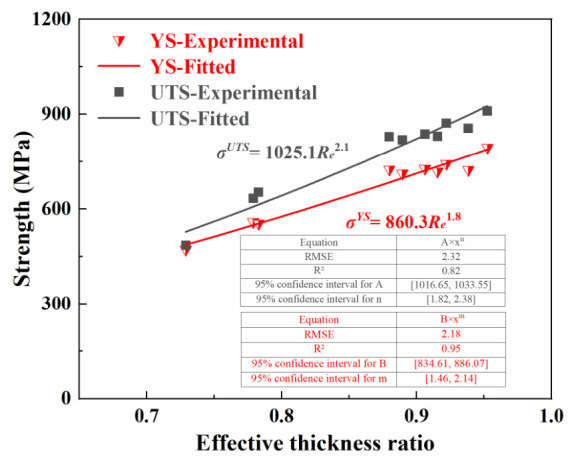
Correlation between strength and effective thickness ratio.

Figure 15 plots the YS and UTS of thin walls with different thicknesses against *R_e_*, where a clear positive correlation can be observed. The corresponding fitted equation is given by

(9) $$\sigma^{YS}=860.3R_{e}^{1.8},\;\sigma^{UTS}=1025.1R_{e}^{2.1}$$

where *σ^YS^* and *σ^UTS^* denote the apparent YS and apparent UTS in the presence of size effects, respectively.

Based on Equation (4), the effective thickness ratio can be calculated quickly, which, when combined with Equation (9), allows for rapid evaluation of the apparent strength for specimens of various thicknesses.

By comparing Equations (5) and (8), it can be seen that:

for YS: *η_s_* = *R_e_*, *η_i_* = *R_e_*^0.8^, *σ*_0_*^YS^* = 860.3 MPa (10a)

for UTS: *η_s_* = *R_e_*, *η_i_* = *R_e_*^1.1^, *σ*_0_*^UTS^* = 1025.1 MPa (10b)

where *σ*_0_*^YS^* and *σ*_0_*^UTS^* denote the theoretical yield strength and ultimate tensile strength of the defect-free Ti-6Al-4V material.

The strength reduction factors of YS and UTS for surface defects are both *R_e_*, while those for coupled interior factors are *R_e_*^0.8^ and *R_e_*^1.1^, respectively. As thickness increases and *R_e_* approaches unity, the reduction effects of the two types become nearly identical. Meanwhile, by dividing Equation (10a) by Equation (10b), the yield-to-tensile ratio of the thin-walled specimen can be derived as 0.84/*R_e_*^0.3^. Within the ultra-thin range, the specimens fail in a brittle manner. Their YS and UTS are relatively close, leading to a higher yield-to-tensile ratio. As the thickness increases, the ratio gradually decreases and converges toward 0.84.

The ultra-thin specimens are highly sensitive to surface defects, making them effective for discerning the influence of such defects on mechanical behavior. For further validation, specimens with thicknesses of 0.2 mm, 0.25 mm, and 0.3 mm were selected and subjected to surface treatment to minimize surface roughness. Manual grinding was performed sequentially using water-lubricated SiC papers from 400 to 2000 grit, followed by polishing with 1 μm alumina suspension, and final ultrasonic cleaning in ethanol. After surface treatment, the apparent thicknesses of the specimens were reduced to 0.135 mm, 0.171 mm, and 0.219 mm, respectively, with approximately 60 μm removed from each sample.

**Figure 16:**
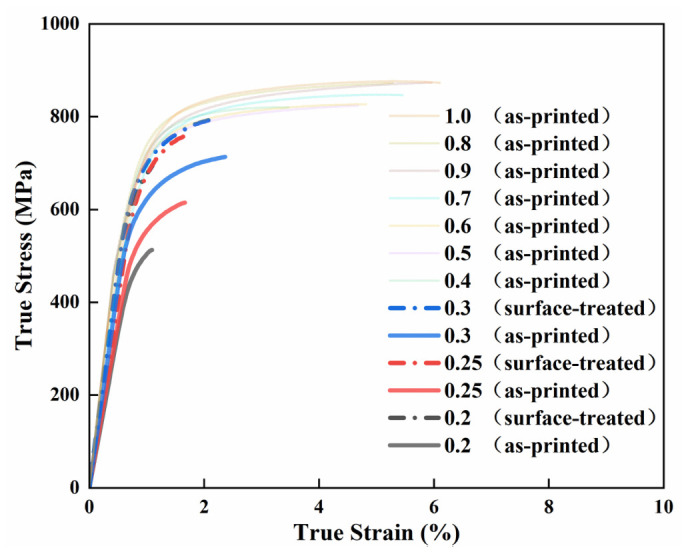
Stress–strain curves of the surface-treated specimens.

The tensile stress–strain curves of the surface-treated specimens are shown in Figure 16. A significant strength improvement is observed compared to their as-printed counterparts. After surface treatment, YS increases from 471.7 ± 19.6–558.9 ± 31.9 MPa to 656.2 ± 11.4–709.6 ± 15.3 MPa, representing a strength increase of approximately 27–39%. UTS rises from 485.2 ± 24.1–652.2 ± 60.8 MPa to 704.2 ± 8.5–796.4 ± 14.6 MPa, corresponding to a growth of about 22–45%. Notably, the overall strength of the surface-treated 0.3 mm specimen approaches that of the as-printed 0.5 mm specimen. However, due to the influence of internal pore defects, its elongation barely changes from the as-printed state. Fracture still occurs at a relatively low elongation, leading to premature failure. Consequently, its strength remains below that of the as-printed 0.5 mm specimen. In the cases of the 0.2 mm and 0.25 mm specimens, internal defects are even more pronounced. Although their strength increases substantially after surface treatment and tends to approach that of the as-printed 0.5 mm specimen, earlier failure induced by these internal defects results in a certain strength gap.

**Figure 17:**
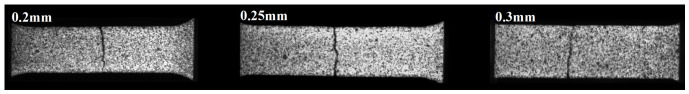
Fracture morphology of the surface-treated specimens.

Figure 17 shows the fracture morphology of the surface-treated 0.2–0.3 mm specimens. Despite the significant reduction in surface defects, the fractures remain transverse to the tensile direction, consistent with the as-printed specimens. This indicates that variations in elongation and fracture mode across different thicknesses are primarily governed by the internal factors, with surface defects playing a relatively minor role.

**Figure 18:**
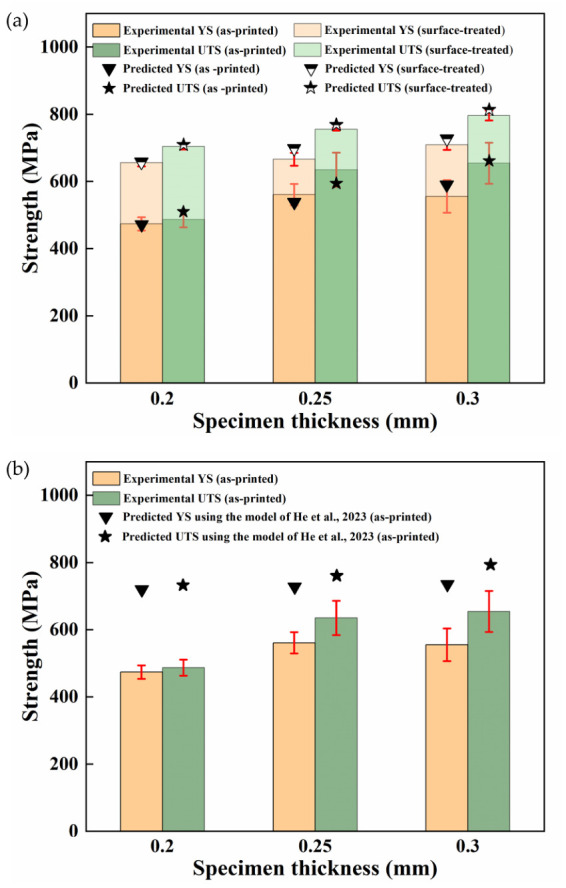
Comparison of experimental and predicted strength using (**a**) the model proposed in this work and (**b**) the model proposed by He et al. [36].

The surface roughness (Ra) values before and after surface treatment are listed in Table 5, showing a decrease from 6.75 to 8.49 μm to 1.53–1.91 μm. This considerable reduction suggests that the rough surface layer was largely removed. Accordingly, the surface weakening effect is assumed to be negligible, allowing *η_s_* to be approximately taken as 1. In this case, the decrease in strength is attributed solely to the coupled interior factors. Therefore, based on Equations (5) and (10), YS and UTS of the ultra-thin specimens were predicted. The predicted results are compared with experimental data in Figure 18a. For further comparison, the empirical model reported in Ref. [36], which was originally valid for thicknesses of 0.5–2.5 mm, was extended to the present ultra-thin range, and the corresponding predictions are shown in Figure 18b.

The predicted strengths from the model proposed in this work agree well with the experimental results, with a maximum relative deviation of 4.8% across all three thicknesses. This further validates the proposed model and confirms its ability to quantify the contributions of surface defects and other factors. In contrast, the model proposed in Ref. [36] significantly overestimates the strength of the ultra-thin specimens. Therefore, it is not applicable to this thickness regime. Moreover, it fails to evaluate the performance of surface-treated specimens.

It should be noted, however, that the present validation is limited to the ultra-thin thickness range (0.2–0.3 mm). Within this specific scope, the model provides a reliable and efficient tool for estimating the size-dependent strength of LPBF-fabricated Ti-6Al-4V thin walls, which is valuable for designing lightweight structures.

**Figure 19:**
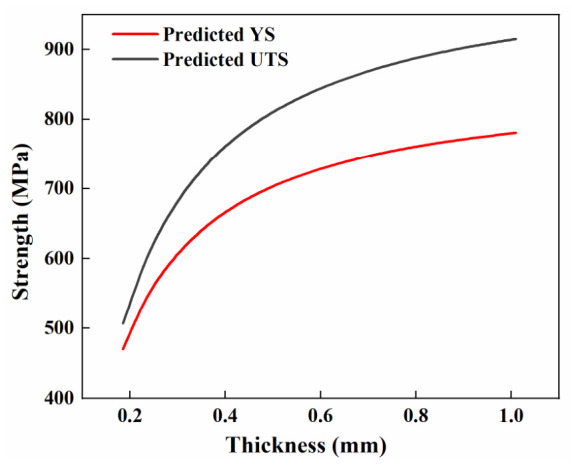
Design guideline for engineering use.

Based on the proposed model, a design guideline figure for engineering use is presented in Figure 19. From this figure, engineers can quickly obtain the predicted strength for a given thickness.

## 4. Conclusions

This study investigated the size effect in Ti-6Al-4V thin walls with thicknesses ranging from 0.2 to 1.0 mm. The variations in strength, elongation, and failure mode with thickness were analyzed. The influencing factors, including microstructure, surface condition, and internal defects, were examined and quantitatively assessed to develop a size-dependent strength-prediction model. The main conclusions are as follows:

LPBF-fabricated Ti-6Al-4V thin walls exhibit a pronounced size effect, characterized by a strengthening trend with increasing thickness. Within the ultra-thin regime (0.2–0.3 mm), the strength rises considerably with thickness, while ductility remains limited, with brittle fracture features present. Beyond this range (0.4–1.0 mm), the increase in strength becomes more gradual, while the elongation improves significantly compared to that of the ultra-thin specimens.

The microstructure shows slight refinement with decreasing thickness. Although this refinement contributes to some strengthening, its role in the overall size effect remains minor. In contrast, surface and internal defects are the key factors governing the size effect in thin walls. Specifically, the strength of the thin walls is controlled by a combination of surface and internal defects, while elongation and fracture behavior are predominantly governed by internal defects. Surface treatment effectively eliminates surface defects and thereby improves the mechanical properties of thin-walled structures. For the 0.2–0.3 mm ultra-thin walls, the YS increases by about 27–39% and the UTS by about 22–45%.

By introducing an effective thickness ratio parameter, a size-dependent strength prediction model was established for as-printed thin walls, enabling quantification of the individual contributions of surface defects and other coupled interior factors. The comparison between experimental and predicted strengths shows excellent agreement, with a maximum relative deviation of only 4.8% for the surface-treated specimens. This model provides a reliable tool for the rapid estimation of YS and UTS, offering a critical foundation for the design of lightweight ultra-thin structures under the influence of size effects. Nevertheless, owing to its semi-empirical basis, the current model is only applicable within the thickness range examined in this work.

## Figures and Tables

**Table 1:** Chemical composition of alloy powder (*ω*/%).

C	H	O	N	Al	Fe	V	Si	Ti
<0.01	0.0048	0.13	0.0086	6.18	0.035	4.07	0.02	Bal.

**Table 2:** Average α′ lath dimensions for specimens with different thicknesses.

Thickness (mm)	Average Length (μm)	Average Width (μm)
0.2	5.065	0.239
0.25	5.109	0.256
0.3	5.419	0.251
0.4	5.499	0.262
0.5	5.471	0.241
0.6	5.430	0.253
0.7	5.454	0.262
0.8	5.539	0.247
0.9	5.501	0.270
1.0	5.685	0.304

**Table 3:** Thickness of the specimens.

Designed Thickness (mm)	Apparent Thickness (mm)	Effective Thickness (mm)	Effective Thickness Ratio (%)
0.2	0.186 ± 0.015	0.136 ± 0.014	72.9 ± 1.3
0.25	0.230 ± 0.019	0.179 ± 0.016	77.9 ± 1.0
0.3	0.279 ± 0.021	0.219 ± 0.024	78.3 ± 2.7
0.4	0.402 ± 0.026	0.354 ± 0.038	87.9 ± 3.9
0.5	0.516 ± 0.031	0.459 ± 0.036	89.0 ± 1.7
0.6	0.640 ± 0.016	0.580 ± 0.024	90.6 ± 1.6
0.7	0.725 ± 0.015	0.664 ± 0.029	91.5 ± 2.1
0.8	0.826 ± 0.023	0.775 ± 0.027	93.8 ± 1.9
0.9	0.908 ± 0.025	0.837 ± 0.046	92.2 ± 2.6
1.0	1.009 ± 0.027	0.961 ± 0.039	95.3 ± 1.4

**Table 4:** Pore area fractions of metallographic samples for various specimen thicknesses.

Thickness (mm)	Pore Size (μm)	Pore Area Fractions (%)
0.2	3–27	0.615
0.25	2–28	0.546
0.3	6–24	0.511
0.4	2–16	0.084
0.5	3–16	0.067
0.6	3–13	0.049
0.7	3–18	0.038
0.8	5–19	0.038
0.9	1–10	0.019
1.0	4–10	0.018

**Table 5:** Surface roughness (Ra) of the specimens.

Thickness (mm)	Ra Before Treatment (μm)		Ra After Treatment (μm)	
	Front Surface	Back Surface	Front Surface	Back Surface
0.2	7.87	1.64	8.33	1.91
0.25	8.49	1.58	6.75	1.53
0.3	7.82	1.78	8.21	1.63

## Data Availability

The original contributions presented in this study are included in the article/Supplementary Material. Further inquiries can be directed to the corresponding author.

## Supplementary Materials

The following supporting information can be downloaded at: https://www.mdpi.com/article/10.3390/ma19101945/s1. Figure S1: Measurement positions of α′ laths for statistical analysis; Table S1: Dimensions of α′ laths in samples with different thicknesses.
