# Supplementary material for: Size-Dependent Mechanical Properties of Additively Manufactured Ti-6Al-4V Thin Walls
Source: Materials (Basel). 2026 May 9;19(10):1945. doi: 10.3390/ma19101945 (PMC13208675; doi:10.3390/ma19101945)
Supplement: Supplementary file 1 [file materials-19-01945-s001.zip › materials-4233633-supplementary.pdf]

Supplementary Materials

Size-Dependent Mechanical Properties of Additive Manufactured Ti-6Al-4V Thin Walls

Tenglong Xie [1], Chao Ding [1,*], Peng Wang [1], Minghao Huang [1], Shenghang Xu [1], Zhen Wang [2] and Huiping Tang [1]

[1] Zhejiang Key Laboratory of Aerospace Metallic Materials, Hangzhou City University, Hangzhou, Zhejiang 310015, China

[2] School of Civil Engineering, Shaoxing University, Shaoxing, Zhejiang 312000, China

To further quantitatively analyze the variation of the microstructure with thickness, the length and thickness of α' laths were measured using ImageJ software based on the BSE images. For each thickness sample, 50 α' laths were randomly selected for measurement, and the selected positions are marked in yellow in Figure S1. The original measurement data are listed in Table S1. Based on the original data, the approximate average microstructure size of each thickness sample can be calculated, which is presented in Table 2 of the main text.

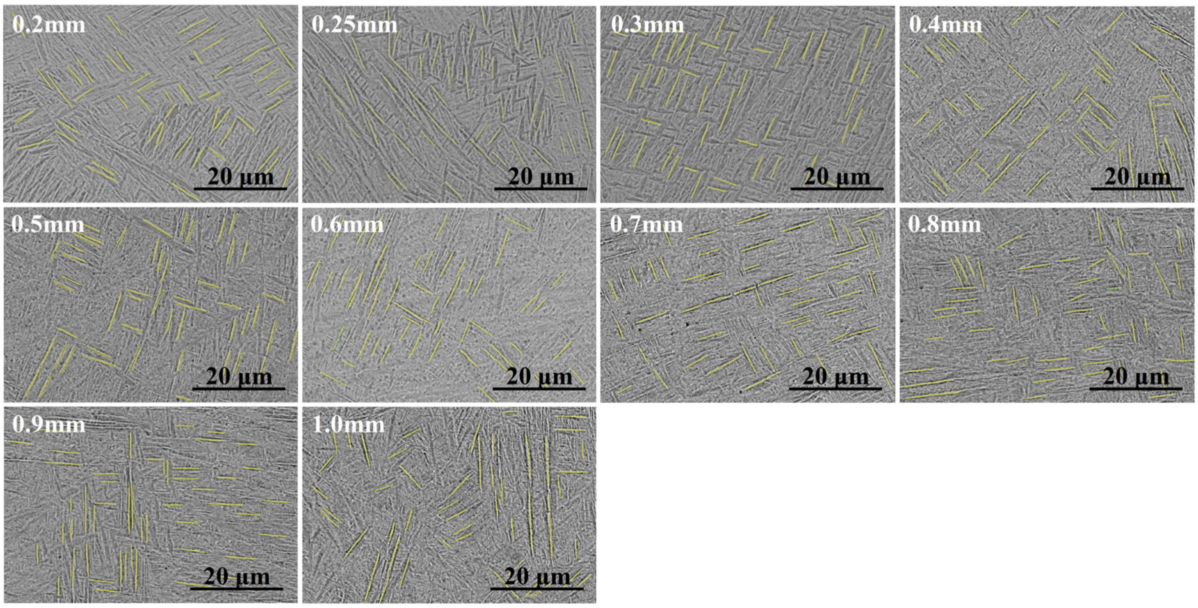

Figure S1. Measurement positions of α' laths for statistical analysis.

Table S1. Dimensions of α' laths in samples with different thicknesses

No.	0.2 mm		0.25 mm		0.3 mm		0.4 mm		0.5 mm	
	Length /μm	Width /μm	Length /μm	Width /μm	Length /μm	Width /μm	Length /μm	Width /μm	Length /μm	Width /μm
1	5.793	0.198	6.710	0.313	3.600	0.292	6.787	0.264	8.200	0.274
2	5.486	0.266	4.364	0.321	3.366	0.255	4.634	0.282	6.014	0.270
3	4.921	0.245	4.485	0.210	6.160	0.290	4.167	0.297	6.014	0.192
4	8.043	0.223	4.667	0.253	6.446	0.259	5.101	0.257	5.360	0.166
5	4.698	0.176	5.140	0.354	5.352	0.248	12.063	0.227	4.895	0.194
6	5.766	0.185	4.762	0.358	5.362	0.230	7.200	0.260	5.312	0.205
7	4.637	0.204	2.767	0.354	4.055	0.246	5.529	0.335	7.501	0.219
8	5.220	0.267	4.144	0.307	5.712	0.170	5.436	0.203	6.675	0.243
9	5.229	0.204	3.966	0.389	4.643	0.234	2.575	0.250	6.619	0.190
10	3.603	0.342	4.711	0.256	6.840	0.296	4.597	0.296	4.341	0.248
11	5.353	0.228	6.021	0.228	4.844	0.258	3.647	0.277	5.057	0.196
12	4.854	0.218	4.292	0.324	5.587	0.243	6.605	0.200	5.674	0.275
13	4.782	0.192	3.367	0.278	5.006	0.253	11.782	0.236	6.542	0.292
14	6.236	0.314	2.870	0.295	6.044	0.194	6.313	0.202	6.368	0.195
15	6.057	0.335	4.786	0.246	7.026	0.178	4.585	0.298	6.954	0.284
16	4.730	0.296	4.773	0.277	9.573	0.210	5.300	0.289	5.029	0.204
17	4.889	0.335	4.360	0.231	6.760	0.127	6.110	0.178	5.846	0.263
18	4.659	0.244	4.427	0.206	3.946	0.136	5.736	0.265	5.057	0.222
19	5.272	0.242	7.132	0.220	5.647	0.239	6.328	0.227	5.025	0.191
20	4.474	0.223	6.076	0.192	6.249	0.208	9.663	0.181	4.977	0.192
21	4.396	0.218	5.876	0.290	4.258	0.200	6.172	0.309	4.656	0.264
22	4.686	0.277	6.511	0.252	5.340	0.242	6.462	0.189	4.919	0.163
23	6.041	0.230	4.685	0.220	6.468	0.233	4.131	0.193	5.011	0.261
24	5.962	0.204	3.537	0.190	6.097	0.356	3.135	0.201	4.928	0.233
25	6.296	0.236	4.075	0.178	6.439	0.253	4.208	0.293	4.924	0.343

26	4.500	0.176	4.037	0.376	4.852	0.261	3.976	0.316	4.766	0.273
27	3.673	0.198	8.620	0.253	5.116	0.212	4.956	0.317	6.424	0.336
28	6.440	0.232	7.296	0.194	3.097	0.410	4.741	0.232	4.662	0.297
29	4.388	0.274	4.655	0.266	7.611	0.414	5.386	0.257	4.615	0.228
30	6.944	0.181	4.432	0.209	7.250	0.231	5.517	0.225	5.183	0.244
31	6.903	0.191	4.757	0.302	5.462	0.275	10.333	0.214	3.157	0.247
32	3.478	0.222	3.901	0.290	4.285	0.234	5.106	0.274	6.534	0.174
33	5.768	0.219	5.934	0.194	5.813	0.290	6.406	0.242	5.562	0.224
34	5.062	0.214	5.731	0.221	4.258	0.253	5.752	0.363	4.347	0.293
35	6.882	0.259	5.532	0.216	4.936	0.178	7.030	0.318	5.293	0.241
36	2.398	0.228	8.935	0.207	3.951	0.216	4.025	0.352	3.770	0.249
37	4.931	0.205	5.234	0.163	3.848	0.191	4.430	0.265	4.851	0.246
38	4.954	0.269	6.298	0.283	6.094	0.330	3.273	0.269	7.072	0.292
39	5.226	0.260	4.590	0.178	5.111	0.277	4.192	0.288	6.527	0.215
40	3.280	0.322	4.837	0.267	4.629	0.365	4.388	0.273	5.230	0.197
41	2.468	0.334	4.454	0.242	5.035	0.296	4.133	0.237	4.778	0.199
42	3.676	0.143	5.650	0.261	4.225	0.248	4.705	0.273	4.664	0.231
43	5.904	0.320	5.266	0.219	4.967	0.215	4.265	0.272	5.173	0.292
44	5.635	0.302	6.631	0.267	5.657	0.317	4.728	0.341	4.955	0.224
45	5.131	0.141	5.127	0.341	4.693	0.198	5.057	0.287	5.132	0.268
46	4.756	0.266	5.319	0.206	5.814	0.323	3.981	0.248	6.410	0.204
47	4.679	0.294	5.946	0.237	6.078	0.255	5.651	0.241	5.073	0.294
48	3.953	0.192	5.071	0.277	5.517	0.233	5.435	0.353	6.908	0.302
49	5.864	0.210	4.127	0.216	6.615	0.143	4.909	0.208	4.738	0.195
50	4.267	0.185	4.543	0.189	5.225	0.359	4.301	0.225	5.829	0.320
**Average**	**5.065**	**0.239**	**5.109**	**0.256**	**5.419**	**0.251**	**5.499**	**0.262**	**5.471**	**0.241**

No.	0.6 mm		0.7 mm		0.8 mm		0.9 mm		1.0 mm	
	Length /μm	Width /μm	Length /μm	Width /μm	Length /μm	Width /μm	Length /μm	Width /μm	Length /μm	Width /μm
1	6.871	0.245	7.680	0.244	11.209	0.248	5.987	0.267	7.023	0.424
2	6.433	0.262	6.928	0.285	12.342	0.218	5.624	0.271	7.382	0.193
3	6.074	0.251	4.011	0.347	4.843	0.250	9.275	0.338	5.520	0.199
4	6.372	0.261	7.121	0.203	5.885	0.187	5.280	0.348	4.304	0.333
5	8.363	0.226	4.066	0.273	4.921	0.167	3.732	0.237	8.340	0.264
6	7.220	0.353	4.998	0.261	5.977	0.243	4.943	0.286	5.561	0.286
7	7.948	0.271	6.632	0.237	6.166	0.318	6.294	0.271	10.186	0.203
8	5.251	0.225	8.520	0.305	6.263	0.261	6.836	0.269	11.935	0.373
9	4.473	0.267	7.405	0.246	5.912	0.282	5.827	0.237	3.419	0.496
10	6.128	0.329	3.746	0.178	6.194	0.305	9.076	0.296	8.940	0.546
11	5.376	0.238	3.854	0.255	6.193	0.282	5.503	0.249	5.123	0.337
12	4.844	0.322	5.142	0.273	5.255	0.379	5.821	0.276	5.516	0.337
13	6.036	0.162	5.812	0.278	7.681	0.224	3.954	0.271	5.588	0.292
14	4.642	0.234	6.669	0.255	6.106	0.242	4.401	0.258	3.996	0.327
15	6.530	0.358	7.056	0.281	6.529	0.245	7.448	0.258	6.710	0.199
16	6.961	0.246	7.207	0.249	4.581	0.212	5.279	0.287	4.942	0.213
17	3.768	0.207	5.018	0.251	4.153	0.175	7.055	0.296	6.130	0.202
18	5.860	0.236	4.674	0.238	4.657	0.207	7.385	0.203	6.531	0.447
19	8.434	0.202	3.336	0.214	3.875	0.214	5.010	0.239	4.256	0.272
20	4.350	0.179	4.156	0.203	5.329	0.205	4.678	0.248	4.816	0.419
21	5.359	0.143	4.762	0.273	6.287	0.262	10.085	0.192	4.379	0.331

22	4.605	0.285	4.733	0.239	3.905	0.246	7.094	0.272	3.721	0.264
23	4.262	0.234	10.011	0.285	5.266	0.245	4.061	0.316	4.708	0.258
24	5.521	0.245	5.859	0.273	5.171	0.224	4.134	0.362	4.556	0.297
25	3.681	0.205	5.187	0.231	3.570	0.307	5.368	0.296	5.131	0.235
26	4.266	0.282	5.357	0.354	4.901	0.208	8.140	0.269	4.047	0.767
27	3.348	0.230	6.112	0.248	4.569	0.205	4.266	0.293	8.477	0.647
28	3.930	0.246	3.716	0.243	4.115	0.223	4.332	0.303	7.950	0.314
29	3.095	0.222	6.112	0.245	4.164	0.289	3.860	0.335	6.070	0.279
30	8.012	0.272	5.340	0.264	3.620	0.316	5.098	0.203	6.310	0.245
31	5.487	0.249	4.075	0.257	4.809	0.223	7.916	0.249	4.840	0.384
32	6.434	0.207	5.787	0.337	4.383	0.190	5.398	0.228	6.710	0.524
33	7.393	0.196	4.826	0.252	3.078	0.355	4.131	0.273	4.613	0.280
34	6.434	0.188	5.544	0.340	4.346	0.282	5.442	0.320	4.806	0.192
35	6.871	0.292	6.307	0.273	4.277	0.243	3.790	0.261	5.146	0.291
36	5.119	0.312	5.767	0.209	5.370	0.306	4.332	0.248	4.618	0.233
37	5.729	0.339	4.534	0.213	3.833	0.217	5.430	0.226	3.996	0.344
38	4.265	0.220	3.854	0.204	4.770	0.203	6.650	0.348	4.258	0.227
39	4.626	0.279	4.690	0.293	6.529	0.150	3.587	0.254	4.412	0.123
40	6.270	0.227	4.887	0.248	6.669	0.261	2.978	0.287	3.773	0.212
41	4.718	0.240	5.692	0.244	6.534	0.261	5.482	0.187	6.143	0.197
42	3.985	0.246	4.992	0.354	5.584	0.254	3.655	0.221	4.933	0.161
43	5.149	0.283	4.914	0.264	5.288	0.243	5.351	0.203	5.336	0.302
44	6.617	0.253	4.496	0.258	5.604	0.298	5.898	0.260	5.131	0.247
45	3.799	0.242	4.899	0.286	4.489	0.153	6.168	0.226	3.684	0.247
46	3.737	0.269	5.187	0.307	6.464	0.224	3.114	0.329	5.031	0.244
47	5.698	0.297	5.154	0.240	7.373	0.263	4.405	0.254	4.588	0.165
48	4.034	0.325	5.392	0.244	5.265	0.307	5.016	0.228	9.846	0.176
49	3.615	0.238	5.333	0.249	6.213	0.303	5.013	0.323	4.122	0.317
50	3.526	0.293	5.167	0.286	6.415	0.230	5.449	0.350	6.715	0.352
**Average**	**5.430**	**0.253**	**5.454**	**0.262**	**5.539**	**0.247**	**5.501**	**0.270**	**5.685**	**0.304**
